# Changes in Adiposity, Muscular Strength, and Functional Capacity Following a National Holiday Break in Chilean Students with Intellectual Disabilities Attending Special-Education Schools

**DOI:** 10.3390/healthcare14193325

**Published:** 2026-10-06

**Authors:** Claudio Farías-Valenzuela, Rafael Kons, Paloma Ferrero-Hernández, Emilio Jofré-Saldía, Cristian Cofré-Bolados, Joaquín Hernández-Soto, Enzo Pavez-Castro, Benjamín Pavez-Pavez, Carlos Poblete-Aro, Josivaldo de Souza-Lima, Andrés Godoy-Cumillaf, Matías Henríquez

**Affiliations:** 1 Escuela de Ciencias de la Actividad Física, el Deporte y la Salud, Universidad de Santiago de Chile (USACH), Santiago 9170022, Chile; cristian.cofre@usach.cl (C.C.-B.); joaquin.hernandez@usach.cl (J.H.-S.); enzo.pavez@usach.cl (E.P.-C.); benjamin.pavez.p@usach.cl (B.P.-P.); 2 Department of Physical Education, Faculty of Education, Federal University of Bahia, Salvador 40170-110, Bahia, Brazil; rafakons0310@gmail.com; 3 Vicerrectoría de Investigación e Innovación, Universidad Arturo Prat, Iquique 1110939, Chile; paloma.ferreroh@gmail.com; 4 Escuela de Ciencias de la Actividad Física, Facultad de Ciencias de la Rehabilitación y Calidad de Vida, Universidad San Sebastián, Santiago 7510141, Chile; ejofres@docente.uss.cl; 5 Escuela de Ciencias de la Actividad Física, Universidad de Las Américas, Santiago 8320000, Chile; cpobletea@udla.cl; 6 Facultad de Educación y Humanidades, Escuela de Ciencias del Deporte, Universidad Andrés Bello, Las Condes, Santiago 7550000, Chile; josivaldo.desouza@unab.cl; 7 Grupo de Investigación en Educación Física, Salud y Calidad de Vida (EFISAL), Facultad de Educación, Universidad Autónoma de Chile, Temuco 4780000, Chile; andres.godoy@uautonoma.cl; 8 Escuela de Kinesiología, Facultad de Salud, Universidad Santo Tomás, Santiago 9170022, Chile

**Keywords:** intellectual disability, adiposity, waist-to-height ratio, handgrip strength, functional capacity, school recess, cardiometabolic risk, special education

## Abstract

Background: National holiday breaks may involve less structured daily routines and cultural practices that favor excess energy intake, yet short-term changes in youth with intellectual disability (ID) following these breaks remain underexplored. This study examined changes in adiposity, muscular strength, and functional capacity following Chile’s national holiday break among students with ID. Methods: In this observational, single-group pre–post study, 49 students with mild-to-moderate ID (35 males, 14 females; 15.9 ± 4.0 years) were assessed before and after the holiday break. Anthropometric and adiposity indicators (body weight, waist [WC] and neck [NC] circumference, BMI, waist-to-height ratio [WHtR], submandibular skinfold [SS]), handgrip strength (absolute, relative) and functional capacity (countermovement jump, five-repetition sit-to-stand, timed up-and-go) were evaluated. Sex-differential changes were assessed using the time-by-sex interaction in mixed-model ANOVA, with Holm correction applied across outcomes within each domain. Within-sex pre–post comparisons were also adjusted using the Holm procedure. Results: No time-by-sex interaction remained significant after correction (all adjusted *p* ≥ 0.137). Significant time effects remained for SS (adjusted *p* = 0.012) and handgrip strength (adjusted *p* < 0.001), but not for NC. In secondary analyses, only the increase in SS (+15.6%; adjusted *p* = 0.025) and the increases in absolute (+8.0%) and relative (+7.6%) handgrip strength in males (both adjusted *p* = 0.002) remained significant. Changes in females were not statistically significant and were therefore interpreted descriptively (all adjusted *p* ≥ 0.31), and no significant changes in functional capacity were detected. Conclusions: The national holiday break was associated with a small increase in SS and an increase in handgrip strength, with the latter potentially reflecting a familiarization effect rather than a true physiological gain in strength. After correction for multiple comparisons, the findings do not support sex-specific conclusions; the observed pattern among females should therefore be considered hypothesis-generating and requires confirmation in larger, controlled studies.

## 1. Introduction

Holiday periods are associated with changes in body composition, as they are characterized by increased energy intake, social gatherings, cultural traditions, and reduced physical activity, all of which have been linked to increased adiposity and weight gain [1,2]. Across many countries, national holiday celebrations are important sociocultural events that preserve culinary, musical, and cultural traditions while promoting social gatherings. Within these festivities, food plays a central role, potentially influencing participants’ eating behaviors and body composition [3,4]. In this context, these celebrations are also associated with behavioral changes, including increased consumption of highly energy-dense foods [5] and increased sedentary behavior [6], both of which are facilitated by periods of festivity, holidays and social gatherings, including family and school communities.

For school-aged children, these annual celebrations often coincide with temporary interruptions to regular school routines and represent culturally important family events that preserve traditions and promote shared experiences during childhood [7]. In Chile and several Latin American countries, these holidays also coincide with a short school recess established by national educational policies, providing students and their families with time away from regular academic activities [8]. During these breaks, students and their families may experience temporary changes in daily routines and health-related behaviors, although the magnitude and nature of these changes may vary across populations and cultural contexts [9]. Such changes may be particularly relevant for children and adolescents with intellectual disability (ID), a neurodevelopmental condition characterized by significant limitations in intellectual functioning (IQ < 70) and adaptive behavior [10,11]. Students with ID attending special educational schools may be particularly vulnerable to the health-related consequences of these temporary disruptions to daily routines [12].

People with ID usually experience a higher prevalence of obesity and lower participation in healthy lifestyle behaviors than their peers without disabilities, showing a combined overweight and obesity prevalence of around 33% [13]. Therefore, monitoring adiposity is an important component of health assessment in this population. Several anthropometric indicators are commonly used to assess adiposity, including body mass index (BMI) and waist circumference (WC) [14], as well as emerging measures such as neck circumference (NC), waist-to-height ratio (WHtR) and submandibular skinfold (SS) [15]. In addition to excess adiposity, reduced muscular strength is another common health concern in people with ID, who exhibit lower levels of voluntary muscle activation [16]. This concern is particularly relevant in the context of premature or accelerated aging in people with ID, which may contribute to an earlier onset of functional decline across the life course [17]. Muscular strength is commonly assessed using absolute (AHGS) and relative (RHGS) handgrip strength [18]. Greater handgrip strength is associated with greater functional capacity [19] and a lower risk of overweight and obesity in people with ID [20]. Functional capacity reflects the physical abilities required for activities of daily living and is closely associated with functional independence [21]. It is commonly assessed using the timed up-and-go (TUG), five-repetition sit-to-stand (5R-STS), and countermovement jump (CMJ) tests [22]. People with ID typically demonstrate reduced performance on these tests, reflecting substantial impairments in muscular strength and functional capacity [23]. However, it remains unclear whether a disruption lasting only one week is sufficient to produce detectable and physiologically meaningful changes in adiposity, muscular strength, or functional capacity, particularly because small changes in anthropometric circumferences may fall within the expected measurement variability.

Evidence regarding health-related changes associated with holiday breaks in school-aged populations remains inconsistent. International studies have reported increases in adiposity indicators, including BMI, WC, and WHtR, whereas changes in physical fitness are less consistent. Moreover, some studies reported stable muscular strength and cardiorespiratory fitness [24], while others showed declines in cardiorespiratory capacity [25]. In Chile, the one-week national break (Fiestas Patrias in Spanish) has been associated with increased BMI and reduced cardiorespiratory fitness in university students [5], although no significant changes in BMI or WC have been observed in adults despite increased caloric intake [26]. However, despite the increased vulnerability of individuals with ID to obesity and poor physical fitness, evidence regarding changes in adiposity, muscular strength, and functional capacity following a brief holiday break remains scarce. Therefore, the present study aimed to examine changes in adiposity indicators, muscular strength, and functional capacity following the national holiday break among Chilean students with ID attending special-education schools and, as a secondary and exploratory aim, to examine whether these changes differed between males and females.

## 2. Materials and Methods

### 2.1. Study Design and Participants

This quantitative, descriptive, observational study used data from the “Ludoinclusión^®^ (2021–2025)” project [22,27]. Participants were recruited through consecutive convenience sampling from three special-education centers in Santiago, Chile. The study sample comprised 49 students with ID (35 males and 14 females) with a mean age of 15.89 ± 4.02 years. Inclusion criteria were: (i) age between 13 and 26 years; (ii) a diagnosis of mild (IQ 50–69) or moderate (IQ 35–49) ID [28], with IQ scores extracted from the internal records of each participating educational center. IQ assessments were conducted by the psychologist of each educational center using the Wechsler Intelligence Scale for Children (WISC-III) [29] for those under 16 years and the Wechsler Adult Intelligence Scale (WAIS-IV) [30] for those aged 16 years or older; (iii) independent mobility; (iv) regular participation in physical-education classes (at least 90 min/week); and (v) the absence of physical and/or sensory disability. Exclusion criteria were: (i) severe or profound ID (IQ < 35) [28]; (ii) requiring assistance to perform motor tasks; (iii) wheelchair use; and (iv) incomplete assessment of adiposity, muscular strength, or functional capacity. The study was conducted in accordance with the ethical guidelines for research involving human beings set out in the Declaration of Helsinki [31] and was approved by a university Ethics Committee (No. 2052/CEIH/2021).

### 2.2. Procedure

**Figure 1:**
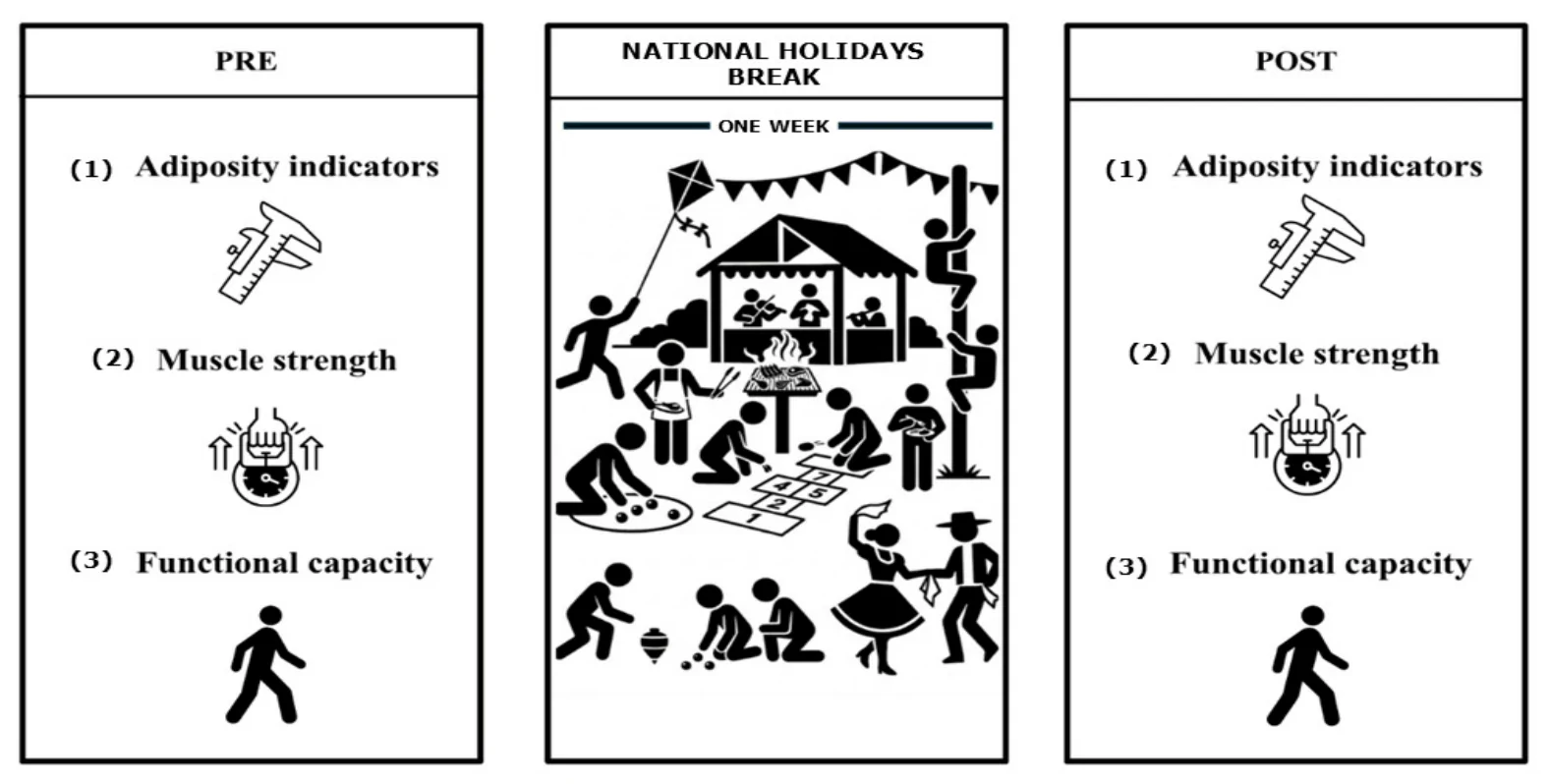
Overview of the three-station assessment circuit (adiposity indicators, muscular strength and functional capacity).

Data were collected in September 2025 at two time points, immediately before and after the Chilean National Holidays (Fiestas Patrias in Spanish) school break, with an 11-day interval between assessments. In August 2025, informational meetings were held outside school hours with the participants’ parents and/or legal guardians, without the presence of the students. During these meetings, the research team explained the study objectives, project phases, procedures, and assessment protocols. Written informed consent was obtained from the parents and/or legal guardians at that time, regardless of whether the participant was an adolescent or an adult. Participation was voluntary and anonymous, and participants had the right to withdraw from the study at any time without consequences. Pre- and post-break assessments were conducted in the morning, at comparable times of day, under standardized conditions with respect to clothing, recent food and fluid intake, bladder status, and prior physical activity. Following the methodology of previous studies [22], participants completed a three-station assessment circuit. Station 1 included the assessment of adiposity indicators, comprising the measurement of body weight, height, WC, NC, SS, BMI and WHtR. Station 2 assessed muscular strength through the AHGS and RHGS handgrip measurements. Finally, station 3 assessed functional capacity by means of the TUG, 5R-STS and CMJ tests (Figure 1).

### 2.3. Adiposity Indicators

Body weight (kg) and height (m) were recorded using a digital scale with an integrated stadiometer (SECA Mod. 769^®^, Hamburg, Germany). Participants were assessed barefoot and wearing light clothing, and height was measured with the head positioned according to the Frankfurt plane [32]. WC (cm) and NC (cm) were measured using an inextensible metal tape measure (CESCORF^®^, Porto Alegre, Brazil). WC was measured at the midpoint between the lower costal margin and the superior iliac crest [33], whereas NC was measured at the midpoint of the neck, at the level of the thyroid cartilage [34]. The SS (mm) was assessed with the participant standing and looking straight ahead, measuring the skinfold located on the line between the thyroid cartilage and the chin in the anteroposterior direction [32]. All anthropometric assessments were performed by the same trained evaluator under standardized conditions. From these anthropometric measurements, BMI and WHtR were calculated from these measurements and used as indicators of cardiometabolic risk [35].

### 2.4. Muscular Strength

Upper-limb muscular strength was assessed by handgrip strength using a hydraulic dynamometer (Baseline^®^, New York, NY, USA), following the protocol established by the American College of Sports Medicine [36] and previously applied in people with ID [19]. The procedure consisted of three attempts per limb [18]. First, a familiarization phase was performed with each arm, followed by two alternating maximal five-second attempts per limb, with a one-minute recovery interval between measurements [22]. The average of the two attempts was taken as the final absolute handgrip strength (AHGS) value for each limb. Relative handgrip strength (RHGS) was then calculated using the formula: RHGS = AHGS (kg)/body weight (kg) [19,37].

### 2.5. Functional Capacity

Functional capacity was assessed using three field tests previously validated in people with ID [22]:

(a) Countermovement jump (CMJ). Participants started the test standing, with their hands on their hips, and then performed an approximately 90° knee flexion followed by a maximal vertical jump while avoiding arm movement [27], using a contact platform (Chronojump^®^, Barcelona, Spain). Before testing, the assessor demonstrated the procedure, and participants completed two familiarization trials. The mean jump height (cm) of two valid attempts was recorded as the final outcome. During testing, foot position was visually monitored, and participants received standardized verbal encouragement to perform each jump with maximal effort.
(b) Five-repetition sit-to-stand test (5R-STS). Participants completed five consecutive sit-to-stand repetitions as quickly as possible from a 43 cm chair. Throughout the test, participants kept their feet in contact with the floor and their arms crossed over the chest, avoiding the use of the upper limbs to assist the movement. The total time required to complete the five repetitions (s) was recorded [38].
(c) Timed Up-and-Go test (TUG). Participants stood up from a chair, walked as quickly as possible around an obstacle positioned 3 m away, turned, returned to the chair, and sat down. Before testing, participants were instructed to begin seated with their back against the backrest and both feet flat on the floor, without using their upper limbs to assist standing. Two familiarization trials were performed, and the time (s) of the valid trial was recorded as the final outcome [39].

**Figure 2:**
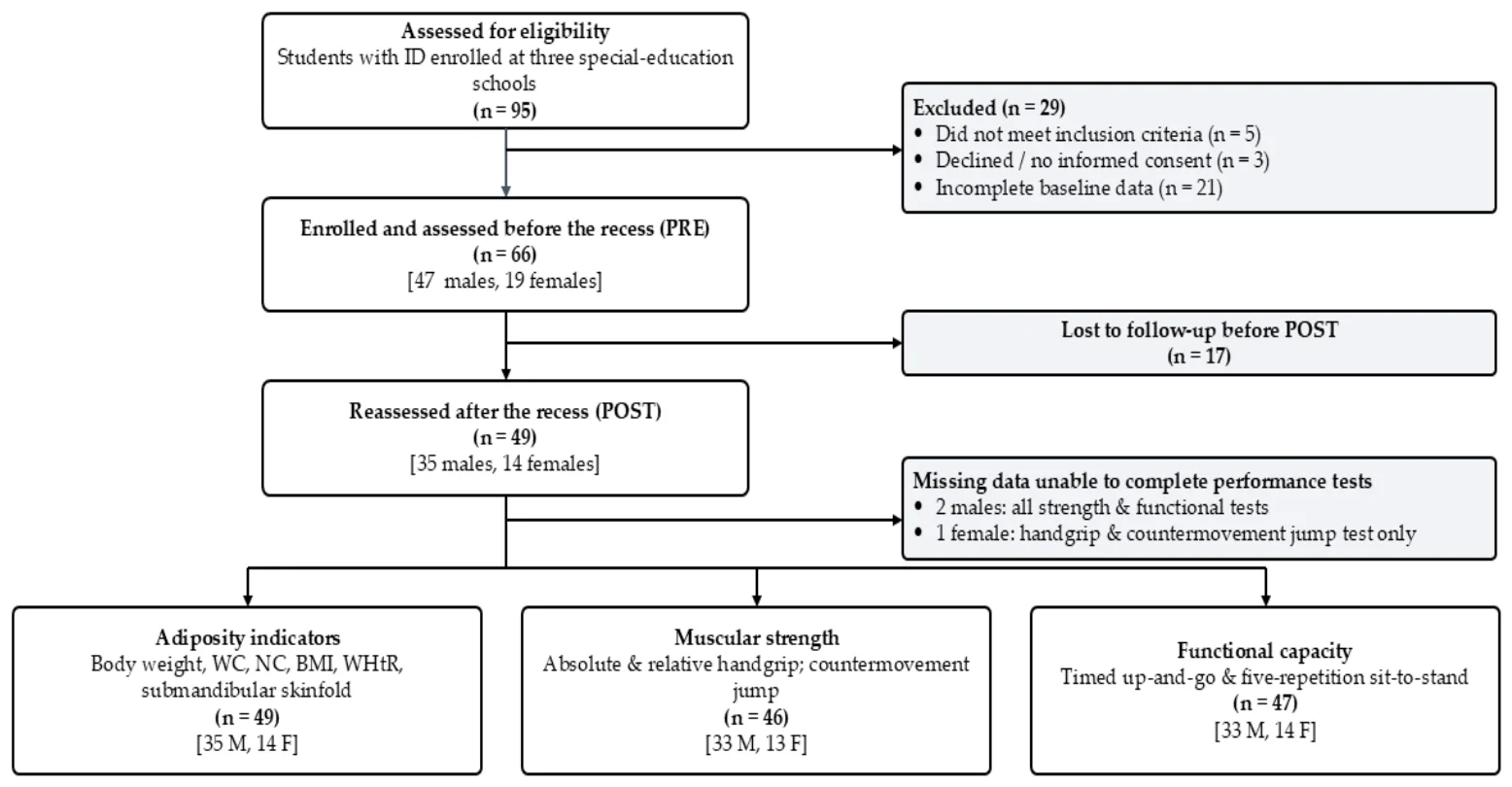
Participant flow diagram. Flow of students with ID through eligibility, pre- (PRE) and post-break (POST) assessment, and inclusion in the adiposity, muscular strength, and functional capacity analyses.

Of 95 students with ID assessed for eligibility, 29 were excluded (5 not meeting inclusion criteria, 3 declining or without informed consent, 21 with incomplete baseline data), leaving 66 participants [47 males, 19 females] assessed before the national holiday break (PRE). After 17 losses to follow-up, 49 participants [35 males, 14 females] were reassessed after the break (POST) and formed the analytic sample. Adiposity indicators were available for all 49; because 3 participants could not complete some performance tests (2 males, all tests; 1 female, handgrip and countermovement jump), muscular strength was analysed in 46 [33 males, 13 females] and functional capacity in 47 [33 males, 14 females] (Figure 2).

### 2.6. Statistical Analysis

Statistical sensitivity was estimated in GPower 3.1 for the sex-stratified analyses (49 participants; 35 males, 14 females), assuming paired comparisons with *α* = 0.05 and Cohen’s *dz* = 0.50. With 80% power, the minimum detectable within-sex effect was *dz* = 0.49 in males (n = 35) and *dz* = 0.81 in females (n = 14), and the power to detect *dz* = 0.50 was 0.82 and 0.41, respectively; for the time-by-sex interaction (equivalent to comparing change scores between sexes), the minimum detectable between-sex difference in change was *d* = 0.91. The study was therefore adequately powered only for medium-to-large within-sex effects in males and for large sex differences in change. Descriptive statistics are presented as mean ± standard deviation (SD) and absolute and percentage delta changes (Δ). For each outcome, a two-factor mixed-model ANOVA (split-plot design) was fitted, treating sex as the between-subjects factor, time (PRE vs. POST) as the within-subjects factor, and participant as the random subject term. Models were estimated by least squares using Type II sums of squares, with partial eta squared (η^2^p) reported as the effect size for the time-by-sex interaction. Because the within-subjects factor had only two levels (PRE, POST), sphericity was satisfied by definition and no covariance-structure correction was required. Model assumptions were evaluated with the Shapiro–Wilk test and visual inspection of the residuals, and outcomes that departed from normality were additionally examined with the corresponding non-parametric within-sex tests. All models were fitted to complete cases (listwise deletion), which explains the differing sample sizes across domains. Unlike a conventional one-factor repeated-measures ANOVA, this design also includes sex as a between-subjects factor and the time-by-sex interaction as the formal test of sex-differential change; the term “mixed-model ANOVA” is used consistently throughout to denote it. To address multiplicity at the omnibus level, the *p*-values for the main effect of time and for the time-by-sex interaction were adjusted using the Holm procedure across the outcomes of each domain (adiposity, muscular strength and functional capacity outcomes). Sex-differential change was inferred exclusively from the Holm-adjusted time-by-sex interaction. Given the observational design and the modest sample, all sex-stratified analyses are considered exploratory. Within-sex pre–post comparisons used paired-samples Student’s *t*-test (parametric variables) or the Wilcoxon signed-rank test (non-parametric variables), with Cohen’s *dz* and its 95% confidence interval (CI) reported as the effect size for all within-sex comparisons, including those analysed with the Wilcoxon test, to allow comparability across outcomes; *dz* was interpreted using conventional thresholds (trivial < 0.20; small 0.20–0.49; medium 0.50–0.79; large ≥ 0.80) [40]. Within-sex *p*-values were adjusted for multiple comparisons using the Holm procedure within each outcome domain (adiposity indicators; muscular strength and functional capacity), with significance defined by the Holm-adjusted *p*-values (A) and unadjusted *p*-values also reported. Analyses were performed in JASP^®^ (version 0.98.1, Amsterdam, The Netherlands), with significance set at *p* < 0.05.

## 3. Results

A total of 49 students with ID participated in the study, including 35 males (24 adolescents and 11 adults; mean age: 16.4 ± 4.08 years) and 14 females (11 adolescents and 3 adults; mean age: 14.79 ± 3.67 years). Descriptive and inferential statistics for anthropometric and adiposity indicators are presented in Table 1, while those for muscular strength and functional capacity outcomes are presented in Table 2.

### 3.1. Anthropometry and Adiposity Indicators

In males, body composition remained largely stable across the break. Body weight (74.09 ± 24.44 to 74.16 ± 24.82 kg; +0.1%), WC (91.62 ± 19.50 to 91.81 ± 19.92 cm; +0.2%), NC (36.83 ± 5.30 to 37.06 ± 5.55 cm; +0.6%), BMI (26.73 ± 7.34 to 26.64 ± 7.41 kg·m^−2^; −0.3%) and WHtR (0.55 ± 0.11 at both time points) exhibited trivial-to-small changes that were non-significant before and after Holm correction (all unadjusted *p* ≥ 0.199; |*dz*| ≤ 0.22). The only anthropometric change in males to remain significant after correction was the increase in SS, which rose from 7.79 ± 5.33 to 9.00 ± 5.89 mm (+15.6%; *p* = 0.002; *p*_(A)_ = 0.025; *dz* = 0.56, medium). In females, small increases were observed in WC, NC, WHtR and BMI; however, none of these within-group changes remained significant after Holm correction and they are therefore reported descriptively rather than as evidence of change. WC increased from 79.39 ± 13.41 to 81.79 ± 11.97 cm (+3.0%; *p* = 0.048; *p*_(A)_ = 0.386; *dz* = 0.58, medium), NC from 31.33 ± 2.34 to 31.88 ± 2.57 cm (+1.8%; *p* = 0.028; *p*_(A)_ = 0.312; *dz* = 0.66, medium), WHtR from 0.510 ± 0.082 to 0.526 ± 0.071 (+3.2%; *p* = 0.035; *p*_(A)_ = 0.353; *dz* = 0.61, medium) and BMI from 22.14 ± 5.18 to 22.35 ± 4.94 kg·m^−2^ (+0.9%; *p* = 0.035; *p*_(A)_ = 0.353; *dz* = 0.49, small). By contrast, body weight (54.30 ± 15.21 to 54.59 ± 14.84 kg; +0.5%; *p* = 0.240; *dz* = 0.33, small) and SS (6.79 ± 2.78 to 7.21 ± 3.40 mm; +6.3%; *p* = 0.435; *dz* = 0.22, small) did not change significantly even before correction. At the omnibus level, nominal time-by-sex interactions were observed for WC (*p* = 0.042; η^2^*p* = 0.085) and WHtR (*p* = 0.023; η^2^*p* = 0.106), with a non-significant interaction for BMI (*p* = 0.092; η^2^*p* = 0.059). However, none of these interactions remained significant after Holm adjustment across the six adiposity outcomes (*p*_(A)_ = 0.209, 0.137 and 0.369, respectively). The main effect of time remained significant after Holm adjustment for SS (*p* = 0.002; adjusted *p*_(A)_ = 0.012), but not for NC (*p* = 0.027; *p*_(A)_ = 0.134). Between-sex main effects reflected the higher baseline adiposity of males for body weight (*p* = 0.008), BMI (*p* = 0.045) and NC (*p* < 0.001). Within-sex estimates, unadjusted omnibus estimates, and Holm-adjusted time-by-sex interaction *p*-values are presented in Table 1.

### 3.2. Muscle Strength and Functional Capacity

Handgrip strength was higher following the holiday break, whereas jump and functional-mobility performance were preserved. In males, AHGS increased from 25.17 ± 11.39 to 27.20 ± 12.66 kg (+8.0%; *p* < 0.001; *p*_(A)_ = 0.002; *dz* = 0.72, medium) and RHGS from 0.36 ± 0.15 to 0.38 ± 0.16 kg·kg^−1^ (+7.6%; *p* < 0.001; *p*_(A)_ = 0.002; *dz* = 0.72, medium); both increases remained significant after Holm correction. Females showed changes in the same direction but of smaller, non-significant magnitude in AHGS (16.73 ± 4.79 to 17.75 ± 5.81 kg; +6.1%; *p* = 0.238; *dz* = 0.34, small) and RHGS (0.311 ± 0.081 to 0.329 ± 0.108 kg·kg^−1^; +6.0%; *p* = 0.217; *dz* = 0.36, small). At the omnibus level, these strength differences corresponded to significant main effects of time for both absolute (*p* < 0.001) and relative (*p* < 0.001) strength, which remained significant after Holm adjustment (both adjusted *p*_(A)_ < 0.001), with no time/sex interaction (*p* = 0.288 and *p* = 0.546, respectively; Holm-adjusted *p*_(A)_ = 0.576 for both); among the handgrip outcomes, a between-sex main effect was present only for AHGS (*p* = 0.013), favouring males. CMJ remained unchanged in both males (17.05 ± 8.50 to 16.67 ± 8.45 cm; −2.2%; *p* = 0.683; *dz* = −0.07, trivial) and females (11.80 ± 4.88 to 11.47 ± 5.50 cm; −2.8%; *p* = 0.632; *dz* = −0.14, trivial), showing only a between-sex difference (*p* = 0.036) with no effect of time or interaction. Functional mobility showed small, non-significant changes: in the TUG, males changed from 6.02 ± 1.57 to 5.97 ± 1.50 s (−0.7%; *p* = 0.666; *dz* = −0.08, trivial) and females from 6.41 ± 1.31 to 6.12 ± 0.92 s (−4.4%; *p* = 0.247; *dz* = −0.32, small), while in the 5R-STS males improved from 9.75 ± 2.75 to 9.35 ± 2.29 s (−4.2%; *p* = 0.223; *dz* = −0.22, small) and females from 9.54 ± 1.50 to 9.28 ± 1.69 s (−2.8%; *p* = 0.461; *dz* = −0.20, small); none of the time, sex or interaction effects reached significance for either test (all *p* ≥ 0.15). None of the female within-group changes in muscular strength or functional capacity reached significance either before or after correction; only the male handgrip gains survived Holm adjustment; however, because the time-by-sex interaction for handgrip strength was not significant, this difference in within-sex significance should not be interpreted as a sex-specific strength response (Table 2).

## 4. Discussion

This study examined changes in the adiposity indicators, muscular strength, and functional capacity among students with ID attending special-education schools following a one-week school holiday break coinciding with Chile’s national celebrations (Fiestas Patrias in Spanish). Three principal findings emerged. First, after correction for multiple comparisons, no time-by-sex interaction remained significant; therefore, the present data do not provide statistical evidence that adiposity changed differently in females and males. Females showed small increases in mean WC, NC, WHtR and BMI, with small-to-medium within-group effect sizes, but none of these changes survived Holm correction, and the nominal interactions for WC and WHtR did not survive adjustment across outcomes. Given also the small female subgroup, the absence of behavioral measurements, and possible measurement variability, this descriptive pattern should be regarded solely as hypothesis-generating and does not establish greater susceptibility to adiposity changes in females with ID. Second, the only adiposity change that was robust to multiplicity control was the increase in SS thickness, supported by both the main effect of time and the within-sex comparison in males. Third, functional capacity (CMJ, 5R-STS, and TUG) remained unchanged in both sexes, whereas handgrip strength was higher after the break (significant main effect of time), with a significant within-sex increase in males only and no time-by-sex interaction.

The small increases observed in some adiposity indicators, of which only the increase in SS was robust to correction for multiple comparisons, would be consistent with the structured days hypothesis, which proposes that the loss of the structured school environment, including supervised routines, scheduled meals, and regular opportunities for physical activity, facilitates obesogenic behaviors, such as excess energy intake, sedentary behavior, and irregular sleep, thereby promoting accelerated weight and fat gain during school breaks [41,42]. Although this hypothesis was originally proposed for longer summer vacations, our findings are compatible with the possibility that a one-week break coinciding with an obesogenic cultural event may be temporally associated with short-term changes in adiposity among students with ID. This interpretation is supported by evidence indicating that dietary habits and food selection are important determinants of body composition in people with disabilities, with greater consumption of energy-dense and processed foods being associated with higher adiposity [43]. Accordingly, temporary changes in eating behavior during holiday periods may plausibly contribute to the short-term anthropometric changes observed in the present study. The Chilean National Holidays are characterized by the consumption of energy-dense traditional foods, and previous studies have reported significant increases in caloric and macronutrient intake during this period, with inconsistent effects on BMI and WC [5,26]. Similarly, body weight did not change significantly in the present study, whereas SS increased and females showed small increases in WC, NC and WHtR that did not survive correction for multiple comparisons; regional adiposity indicators may therefore be worth examining in adequately powered studies as markers of short-term changes that may not be captured by body weight alone. However, because anthropometric circumferences may also be influenced by factors such as hydration status, recent food intake, and measurement variability, these findings should not be interpreted as definitive evidence of rapid fat accumulation. Nevertheless, WC, WHtR, and NC are commonly used as screening indicators of central adiposity and cardiometabolic risk [44,45]. Mean WHtR values were already above 0.50 before the school holiday break in both sexes, suggesting an elevated baseline risk profile rather than a transition to a higher-risk category during the holiday period. Nevertheless, the applicability of pediatric cut-off points to participants older than 18 years should be considered.

Beyond statistical significance and effect-size classifications, the absolute magnitude of these changes also warrants consideration when appraising their clinical and practical relevance. In females, the observed increases (which were not statistically significant after correction for multiple comparisons) corresponded to approximately 2.4 cm in waist circumference and 0.2 kg/m^2^ in BMI; their clinical relevance over such a short period remains uncertain. Changes of this order lie within, or close to, the measurement error typically reported for WC, so short-term differences of this magnitude cannot always be reliably distinguished from measurement variability [46]. In addition, given the wide age range of the sample, spanning adolescence to early adulthood, part of these differences may also reflect normal growth and day-to-day biological fluctuation rather than a clinically meaningful change in adiposity. This developmental heterogeneity may also contribute to variability in muscular strength, as age-related differences in anthropometric characteristics and muscular strength have been reported across children, adolescents, and adults with ID [20]. Therefore, the changes observed should be interpreted cautiously, particularly when comparing participants across different developmental stages. Accordingly, although WC and related indices remain useful markers of cardiometabolic risk [47], the present short-term changes should be interpreted as exploratory signals rather than as evidence of a clinically important change over such a brief period.

Even small changes would be relevant among individuals with ID, a population with a substantially higher prevalence of overweight, obesity, and sedentary behavior than the general population [48,49]. Although the descriptive pattern observed in females would be consistent with evidence that females with ID are more likely to be physically inactive and to develop morbid obesity [50], the absence of a significant time-by-sex interaction after correction for multiple comparisons precludes any inference of sex-specific susceptibility in the present sample.

Nevertheless, dietary intake, physical activity, sleep, and pubertal status were not assessed, and the mechanisms that could underlie any sex-specific pattern remain uncertain. Consequently, the present data can neither confirm nor exclude sex differences in the response to the holiday break: the nominal interactions should not be read as evidence of a sex-specific effect, and the non-significant adjusted interactions should not be read as evidence of equivalent responses. In Chilean students with ID assessed before and after the COVID-19 lockdown using the same battery of anthropometric, strength, and functional measures, the disruption of regular school routines was associated with unfavorable changes in body composition and physical fitness [27]. Although the COVID-19 lockdown differs substantially from a brief festive break, both studies suggest that temporary interruptions to structured daily routines may contribute to unfavorable health-related changes in students with ID. Because many children and adolescents with ID rely on structured environments to support healthy daily routines, including regular meals and opportunities for physical activity, maintaining these routines during school holidays may support healthy lifestyle behaviors [51].

In contrast to the anthropometric findings, functional performance remained unchanged in both sexes. Although males showed higher mean AHGS and RHGS following the school holiday break, the physiological significance of this finding remains uncertain. In the mixed-model analysis, this corresponded to a significant main effect of time for both AHGS and RHGS, with no significant time-by-sex interaction. A true increase in muscular strength over such a short period appears unlikely, as neuromuscular adaptations generally require several weeks of progressive resistance training [52]. Moreover, previous studies have reported that even structured, multi-month adapted physical activity interventions often fail to produce significant improvements in handgrip strength in children and adolescents with ID [53,54]. Several methodological factors may provide a more plausible explanation for the observed increase. Individuals with ID frequently exhibit lower voluntary muscle activation, resulting in greater variability during maximal-effort tests [16]. In addition, handgrip dynamometry is sensitive to testing procedures, participant motivation, and familiarization [55]. Although a familiarization trial preceded each test session, learning effects across sessions were not quantified in the present study; therefore, a learning effect cannot be excluded and may have contributed to the observed changes. Alternatively, if physical activity decreased during the holiday break, a reduction in accumulated fatigue might have contributed to higher handgrip strength at follow-up [56].

The distribution of the data further supports this interpretation. Although mean AHGS and RHGS values increased, the variability across participants suggests that these changes may not reflect a consistent improvement at the group level. Furthermore, no concurrent changes were observed in the CMJ, 5R-STS, or TUG tests. While these assessments measure different aspects of physical function and therefore cannot directly confirm changes in handgrip strength, the absence of improvements across complementary functional measures provides limited support for a meaningful physiological adaptation. Overall, these findings suggest that the increase in handgrip strength observed in males should be interpreted with caution and is more likely attributable to familiarization effects and measurement variability than to a true gain in muscular strength. Existing normative data for handgrip strength and functional performance in Chilean students with ID provide additional context for interpreting the magnitude and direction of these findings [22,37].

From a public health perspective, these findings highlight the potential importance of maintaining structured routines during school breaks, including festive periods, to preserve opportunities for regular physical activity and other healthy lifestyle behaviors among students with ID [57]. Such strategies should aim to preserve the cultural and social value of these celebrations while encouraging healthy eating habits, regular meal patterns, adequate sleep, and accessible opportunities for physical activity. In addition, routine monitoring of simple anthropometric indicators, such as WC and WHtR, may help schools and families identify short-term changes in central adiposity that may not be detected by body weight alone. Several limitations should be considered when interpreting these findings. First, the sample was modest, particularly among female participants (n = 14), limiting statistical power and the precision of the effect size estimates. Second, the wide age range, encompassing adolescents and young adults, may have introduced developmental heterogeneity that could influence anthropometric characteristics, muscular strength, and functional capacity. This heterogeneity may have reduced comparability across developmental stages and should therefore be considered when interpreting and generalizing the findings. Third, the single-group pre–post design without a comparison group does not allow changes potentially associated with the recess to be distinguished from natural short-term biological variability, measurement error, regression to the mean, or other concurrent factors. Fourth, the use of convenience sampling and the inclusion of students with mild-to-moderate ID may limit the generalizability of the findings to individuals with more severe levels of ID, additional disabilities, or different educational and geographical settings. In addition, dietary intake, physical activity, sedentary behavior, and sleep were not assessed; therefore, their potential contribution to the observed changes remains hypothetical. Furthermore, a large number of outcomes were examined in both sexes. Although the Holm procedure was applied to both the within-sex comparisons and the omnibus mixed-model effects, the definition of the testing families by outcome domain is a methodological choice, and the study was neither designed nor powered for confirmatory testing of sex differences; all sex-stratified findings should therefore be regarded as exploratory. Finally, the apparent increase in handgrip strength may have been influenced by reduced accumulated fatigue following the one-week break, as well as measurement variability or familiarization effects, and the inclusion of participants from special education centers within a single city may limit the generalizability of the findings. Therefore, these results should be considered hypothesis-generating. Future studies with larger and more diverse samples, standardized anthropometric assessments, and objective measures of lifestyle behaviors are needed to confirm these findings and clarify the mechanisms underlying short-term changes in adiposity during school holiday break. Despite these limitations, and given the uncontrolled pre–post design and the possibility of measurement error, the present findings should be regarded as exploratory and can, at most, suggest that even a short school holiday may be associated with small unfavorable changes in some adiposity indicators, without evidence of sex-specific effects, among students with ID. These results underscore the importance of implementing family- and school-based strategies to promote healthy eating habits, maintain regular physical activity, reduce sedentary behaviors, and preserve daily routines during holiday periods. Such approaches may help mitigate unfavorable changes in health associated with school holiday breaks and contribute to healthier lifestyles in this vulnerable population.

## 5. Conclusions

In this single-group, observational pre–post study, a one-week national holiday break (Fiestas Patrias in Spanish) was associated with a small increase in SS thickness and higher handgrip strength, while no significant changes in functional capacity were observed. The handgrip increase may reflect familiarization or measurement variability rather than a true strength gain. After correction for multiple comparisons, no time-by-sex interaction remained significant, and the observed increases in adiposity among females did not survive correction. Thus, the findings do not support sex-specific conclusions. Larger, controlled studies with pre-specified sex-stratified hypotheses are needed to clarify whether school-break periods differentially affect health-related outcomes in students with ID.

## Figures and Tables

**Table 1:** Sex-specific changes in anthropometric and adiposity indicators before and after the national holiday break in Chilean students with intellectual disability.

Variable	Sex	PRE (M ± SD)	POST (M ± SD)	Δ	Δ%	*dz* (95% CI)	Within-Group (POST vs. PRE)		Mixed-Model ANOVA					
							*p*-Value	(A) *p*-Value	Time *p*-Value	Time (A) *p*-Value	Sex *p*-Value	T × S *p*-Value	T × S (A) *p*-Value	η^2^p (T × S)
Body weight (kg)	M	74.09 ± 24.44	74.16 ± 24.82	+0.07	+0.1%	0.07 (−0.27, 0.42)	0.663	1.000	0.315	0.629	0.008 *	0.442	0.733	0.013
	F	54.30 ± 15.21	54.59 ± 14.84	+0.29	+0.5%	0.33 (−0.26, 0.92)	0.240	1.000						
WC (cm)	M	91.62 ± 19.50	91.81 ± 19.92	+0.19	+0.2%	0.06 (−0.28, 0.41)	0.716	1.000	0.094	0.374	0.056	0.042 *^,^ᵃ	0.209	0.085
	F	79.39 ± 13.41	81.79 ± 11.97	+2.40	+3.0%	0.58 (−0.04, 1.21)	0.048 ᵃ	0.386						
NC (cm)	M	36.83 ± 5.30	37.06 ± 5.55	+0.23	+0.6%	0.22 (−0.13, 0.57)	0.199	1.000	0.027 ᵃ	0.134	<0.001 *	0.305	0.733	0.022
	F	31.33 ± 2.34	31.88 ± 2.57	+0.55	+1.8%	0.66 (0.02, 1.30)	0.028 ᵃ	0.312						
BMI (kg/m^2^)	M	26.73 ± 7.34	26.64 ± 7.41	−0.09	−0.3%	−0.15 (−0.50, 0.19)	0.968 ^†^	1.000	0.962	0.962	0.045 *	0.092	0.369	0.059
	F	22.14 ± 5.18	22.35 ± 4.94	+0.21	+0.9%	0.49 (−0.12, 1.10)	0.035 ^†,^ᵃ	0.353						
WHtR	M	0.55 ± 0.11	0.55 ± 0.11	+0.00	+0.0%	0.00 (−0.34, 0.34)	0.622 ^†^	1.000	0.143	0.429	0.267	0.023 *^,^ᵃ	0.137	0.106
	F	0.51 ± 0.08	0.53 ± 0.07	+0.02	+3.2%	0.61 (−0.01, 1.24)	0.035 ^†,^ᵃ	0.353						
SS (mm)	M	7.79 ± 5.33	9.00 ± 5.89	+1.22	+15.6%	0.56 (0.19, 0.93)	0.002	0.025 *	0.002 *	0.012 *	0.377	0.244	0.733	0.029
	F	6.79 ± 2.78	7.21 ± 3.40	+0.43	+6.3%	0.22 (−0.37, 0.80)	0.435	1.000						

WC = waist circumference; NC = neck circumference; BMI = body mass index; WHtR = waist-to-height ratio; SS = submandibular skinfold; M = male; F = female; M ± SD = mean ± standard deviation; Δ = absolute change POST-PRE; Δ% = percentage change (POST-PRE)/PRE × 100; *dz* = Cohen’s dz for the within-group change with 95% CI. Within-group (PRE vs. POST) *p* unadjusted = paired-samples *t*-test by sex (^†^ Wilcoxon signed-rank test). *p* adjusted (A) = the same *p*-values after Holm correction. Time, Sex and T × S (Time × Sex) *p*-values and η^2^p (partial eta squared) are from a mixed-model ANOVA and are unadjusted; Time (A) and T × S (A) = the corresponding Holm-adjusted *p*-values across the six adiposity outcomes. Holm correction of within-group *p*-values was applied across 12 tests (6 outcomes × 2 sexes). ᵃ = nominally significant before correction (*p* < 0.05) but NOT after Holm adjustment. * = *p* < 0.05.

**Table 2:** Sex-specific changes in muscular strength and functional capacity before and after the national holiday break in Chilean students with intellectual disability.

Variable	Sex	PRE (M ± SD)	POST (M ± SD)	Δ	Δ%	*dz* (95% CI)	Within-Group		Mixed-Model ANOVA					
							(POST vs. PRE)							
							*p*-Value	(A) *p*-Value	Time *p*-Value	Time (A) *p*-Value	Sex *p*-Value	T × S *p*-Value	T × S (A) *p*-Value	η^2^p (T × S)
AHGS (kg)	M	25.17 ± 11.39	27.20 ± 12.66	+2.02	+8.0%	0.72 (0.32, 1.12)	<0.001	0.002 *	<0.001 *	<0.001 *	0.013 *	0.288	0.576	0.026
	F	16.73 ± 4.79	17.75 ± 5.81	+1.02	+6.1%	0.34 (−0.28, 0.97)	0.238	1.000						
RHGS (kg·kg^−1^)	M	0.36 ± 0.15	0.38 ± 0.16	+0.03	+7.6%	0.72 (0.33, 1.12)	<0.001	0.002 *	<0.001 *	<0.001 *	0.271	0.546	0.576	0.008
	F	0.31 ± 0.08	0.33 ± 0.11	+0.02	+6.0%	0.36 (−0.26, 0.99)	0.217	1.000						
CMJ (cm)	M	17.05 ± 8.50	16.67 ± 8.45	−0.38	−2.2%	−0.07 (−0.43, 0.28)	0.683	1.000	0.598	0.598	0.036 *	0.977	1.000	0.000
	F	11.80 ± 4.88	11.47 ± 5.50	−0.33	−2.8%	−0.14 (−0.74, 0.47)	0.632	1.000						
TUG (s)	M	6.02 ± 1.57	5.97 ± 1.50	−0.04	−0.7%	−0.08 (−0.43, 0.28)	0.666	1.000	0.246	0.492	0.544	0.262	0.786	0.028
	F	6.41 ± 1.31	6.12 ± 0.92	−0.28	−4.4%	−0.32 (−0.92, 0.27)	0.247	1.000						
5R-STS (s)	M	9.75 ± 2.75	9.35 ± 2.29	−0.41	−4.2%	−0.22 (−0.57, 0.14)	0.223	1.000	0.156	0.469	0.837	0.803	1.000	0.001
	F	9.54 ± 1.50	9.28 ± 1.69	−0.27	−2.8%	−0.20 (−0.79, 0.38)	0.461	1.000						

AHGS = absolute handgrip strength; RHGS = Relative handgrip strength; CMJ = Countermovement jump; TUG = timed up and go; 5R-STS = five-repetition sit-to-stand test; M = male; F = female; M ± SD = mean ± standard deviation; Δ = absolute change POST-PRE; Δ% = percentage change (POST-PRE)/PRE × 100; *dz* = Cohen’s *dz* for the within-group change with 95% CI. Within-group (PRE vs. POST) *p* unadjusted = paired-samples *t*-test by sex (Wilcoxon signed-rank test). *p* adjusted (A) = the same *p*-values after Holm correction. Time, Sex and T × S (Time × Sex) *p*-values and η^2^p (partial eta squared) are from a mixed-model ANOVA and are unadjusted. Time (A) and T × S (A) = the corresponding Holm-adjusted *p*-values across the outcomes of each domain (muscular strength: AHGS and RHGS; functional capacity: CMJ, TUG and 5R-STS). Holm correction of within-group *p*-values was applied across 10 tests (5 outcomes × 2 sexes). * = *p* < 0.05.

## Data Availability

The datasets used and/or analyzed during the current study are available from the corresponding author on reasonable request.
